# Frequency of Agenesis Palmaris Longus through Clinical Examination - An East African Study

**DOI:** 10.1371/journal.pone.0028997

**Published:** 2011-12-13

**Authors:** James W. M. Kigera, Stephen Mukwaya

**Affiliations:** 1 Department of Orthopaedics, College of Health Sciences, Makerere University, Kampala, Uganda; 2 Orthopaedics Rehabilitation Unit, PCEA Kikuyu Hospital, Kikuyu, Kenya; 3 Mulago Paramedical Schools, Kampala, Uganda; University of Illinois at Champaign-Urbana, United States of America

## Abstract

**Introduction:**

The Palmaris longus, one of the most variable muscles in the body both flexes the wrist and tenses the palmar fascia. It is used by surgeons as a source of tendon graft and racial differences in its variation have been documented. We sought to determine the frequency of the absence of the Palmaris longus in an East African population.

**Methods:**

A prospective study was conducted using ten common clinical tests among patients and students in a large teaching hospital in East Africa to determine the presence of a Palmaris longus.

**Results:**

The overall rate of absence was 4.4% with unilateral absence at 3.3% and bilateral absence at 1.1%. The overall difference between males and females was not statistically significant (p = 0.605). Participants were more likely to have absence in their non dominant hand.

**Discussion:**

Our findings though in contrast to many studies worldwide, it concurs with most studies done in the African setting. These differences may be due to the higher levels of manual labour and the more use of the right hand in these activities. The frequency of the absence of Palmaris longus in East Africa has been determined. Surgeons should acquaint themselves with prevalence in their areas of practice.

## Introduction

The Palmaris longus (PL) is a small vestigial forearm muscle. The muscle is one of the most variable muscles in the body, varying in position, having duplication and containing accessory slips. Numerous studies have documented that the prevalence of the muscle varies across races [1], [2], [3]. The muscle is often used by hand surgeons as a tendon graft and has been used to reconstruct ligaments and tendons. The Palmaris longus is a weak flexor of the wrist joint and also tenses the palmar fascia[4].Though the rate of absence of the Palmaris Longus has been documented in many African populations, this has not been documented in East Africa by clinical methods in a large population[5], [6]. The Palmaris longus is also of interest because of the association with clinical conditions of the hand, genetics and anthropology [5], [7]. Our goal was to determine if the frequency of the absence of the Palmaris longer was different from other reported populations in Africa and the rest of the world. We also aimed at noting any relationship with sex and dominant hand. These findings will document the frequency of agenesis in this population and will be useful to surgeons working in this particular population.

## Methods

We conducted a prospective study of 800 subjects who were students of the paramedical and nursing schools and patients attending the orthopaedic surgical outpatient clinic. Participants were recruited consecutively from these groups and the conduct of the study explained before informed consent was taken. The subjects were evaluated with 10 tests to detect the presence of the Palmaris Longus. To be considered to have an absence of a Palmaris longus, the subject must have a negative test for all 10 tests. If a subject had a positive result for any of the 10 tests, the subject was considered to have a Palmaris longus. These tests have been previously described. In Schaeffer's test, volunteers were made to steady their forearm at 90° before opposing the thumb to the little finger with the wrist partially flexed[8]. Mishra's 1^st^ test involved passive hyperextension of the metacarpophalangeal joints along with mild active flexion of the wrist[9]. In Thompson's test, a fist was made followed by flexing the wrist against resistance with the thumb flexed over the fingers[10]. In Pushpakumar's “two-finger sign” method, the subjects were made to fully extend the index and middle finger while the wrist and other fingers were fully flexed with the thumb opposed and flexed[11]. The Gangata test involves the subject resisting both thumb abduction and wrist flexion[5]. In Mishra's 2^nd^ test, the subjects were asked to abduct the thumb against resistance with the wrist partially flexed[9]. The four finger sign is a combination of forced opposition and flexion of the thumb at the first metacarpophalangeal joint with full extension of the second to fifth digits[12]. The Lotus sign is done by bringing the fingers and thumb together to form a cone while in the open hand method, the patient is asked to fan out all the fingers and slightly flex the wrist. The Bhattacharya test is performed by having the patient flex their wrist against resistance[13].

Patients with obvious hand and wrist deformities, previous hand and wrist injuries and previous surgery to the hand and/or wrist were excluded. Participants provided written informed consent and assent was sought from the next of kin in the case of those aged below 18 years. The study was approved by the Mulago Hospital ethics board in Kampala Uganda and permission was granted by the paramedical school authorities.

Data was collected by a questionnaire and entered into Epidata program and exported to SPSS v 11.5 (SPSS Inc., Chicago, Illinois). Statistical tests used were the chi square tests for categorical data and the t test for continuous data.

## Results

We examined 800 subjects, the majority (76.1%) of whom were students and right handed (94.4%). There were 391 (48.9%) males and 409 (51.1%) females. The subjects' ages ranged from 12 to 70 years with a mean age of 25 years.

The overall prevalence of the Palmaris longus was 95.6% giving an absence rate of 4.4% with unilateral absence at 3.3% and bilateral absence at 1.1% (Table 1). Right handed people were more likely to have PL agenesis on the non dominant side while left handed people were more likely to have agenesis on the dominant side (Table 2). These differences, however did not reach statistical significance (p = 0.309)

**Table 1:** Overall Absence.

Absence In Right Side	Absence in Left Side	Absence Bilaterally	Total Absence
8 (1.0%)	18 (2.3%)	9 (1.1%)	35 (4.4%)

Table showing the overall absence of the Palmaris longus muscle in the study.

**Table 2:** Overall PL absence by hand dominance.

Absence of PL	Right Handed (%)	Left Handed (%)
Present Bilaterally	723 (95.8)	38 (95.0)
Absent on Right Side	7 (0.9)	0 (0)
Absent on Left Side	17 (2.3)	1 (2.5)
Absent Bilaterally	8 (1.1)	1 (2.5)

Table showing the distribution of agenesis of the PL by hand dominance of the subjects.

In males the overall absence was 4.9% with unilateral absence being 1.0% on the right and 2.6% on the left side. The bilateral prevalence of absence was 1.3%. In females the overall prevalence was 3.9% with unilateral absence at 3.0% and bilateral at 1.0%. The prevalence of absence in the dominant hand was 1.0% while in the non dominant hand it was 2.0% (Table 3).

**Table 3:** Distribution of Palmaris Longus Absence.

Absence of PL	Right	Left	Dominant	Non Dominant	Bilaterally	Total
Male	4 (1.0%)	10 (2.6%)	4 (1.0%)	10 (2.6%)	5 (1.3%)	19 (4.9%)
Female	4 (1.0%)	8 (2.0%)	4 (1.0%)	8 (2.0%)	4 (1.0%)	16 (3.9%)

Table showing the distribution of the absence of the Palmaris longus between males and females and according to the hand dominance.

The overall difference between males and females in terms of absence of a Palmaris longus was not statistically significant (p = 0.605).

## Discussion

Absence of the Palmaris Longus in this study was 4.4%. This differs with the values as high as 25% seen in various Caucasian population studies[3], [14]. This muscle has been reported to have wide variation with absence being reported in 4.5% of the Chinese population, 37.5% of the Serbian population and 63.9% of the Indian population[15], [16], [17], [18].

Various studies done in African populations have shown a lower rate of absence ranging from 6.7% in the Yoruba to 1.5% in Zimbabwe[5], [6]. Our study has also demonstrated this lower rate of absence. It may be that the low rate of absence of this muscle is explained by the higher prevalence of manual labour in African populations. It is plausible to expect wrist motion in manual labour hence increasing instances in which the Palmaris longus is called into action. Manual labour also requires a strong grip which is aided by a tensed palmar fascia. All this may lead to the muscle undergoing less degeneration.

Our study although not statistically significant also shows a trend of participants being more likely to have absence in the non dominant hand when compared to the dominant hand. This is in variance to a study by Eric et al [19]. This finding may be explained by the fact that the dominant hand is more involved in manual activities and hence less likely to degenerate due to disuse as compare to the non dominant hand.

This study informs surgeons working in this region their likelihood of finding the Palmaris longus tendon for tendon grafts or various reconstructive procedures. It has been suggested that the median nerve is more vulnerable in the absence of the Palmaris longus and hence in this population, injuries to the nerve at the wrist could potentially be fewer.

One of the weaknesses of this study was that the presence of a Palmaris longus was determined by clinical exam which can be examiner dependent as compared with a cadaveric study where presence of a Palmaris longus would be determined by visualizing the actual muscle. Nevertheless, one of the strengths of this study was in the use of numerous tests to detect the presence of Palmaris longus, thus increasing the likelihood that all tendons that crossed the wrist were detected. Strength of the study is the large sample size.

There is a low frequency of agenesis of the Palmaris Longus in the East African Population which supports the racial differences previously noted. It is necessary for surgeons in every region to acquaint themselves with the local frequency of agenesis before planning on utilizing this tendon for grafting or other reconstructive purposes.
